# Tensile Properties of <111>-Oriented Nanotwinned Cu with Different Columnar Grain Structures

**DOI:** 10.3390/ma13061310

**Published:** 2020-03-13

**Authors:** Yu-Jin Li, King-Ning Tu, Chih Chen

**Affiliations:** 1 Department of Materials Science and Engineering, National Chiao Tung University, Hsinchu 30010, Taiwan; r777719982003@yahoo.com.tw; 2 Department of Materials Science and Engineering, University of California at Los Angeles, Los Angeles, CA 90095, USA; kntu@ucla.edu; 3 International College of Semiconductor Technology, National Chiao Tung University, Hsinchu 30010, Taiwan

**Keywords:** nano-twinned Cu, tensile properties, grain microstructures

## Abstract

We performed tensile tests on highly <111>-oriented nanotwinned copper (nt-Cu) foils with different columnar grain structures. For a systematic study, we altered the microstructure of the foils by tuning the electroplating electrolyte and annealing temperatures under a nitrogen atmosphere. The results show that the yield strength ranges from 300 to 700 MPa, and elongation spans from 5% to 25%. Knowing the measured twin spacing and average grain size, and combining the confined layer slip with the Hall–Petch equation, we calculated the theoretical yield strength of the nt-Cu with different microstructures, and the theoretic values match the experiment results. Owing to the unique crystal orientation properties of <111>-oriented columnar grains, dislocations induced by slip are very limited. The Schmid factor of grains along the tensile axis direction is highly identical, so the plastic deformation is much more suitably explained by the Schmid factor model. Thus, we replace the Taylor factor with the Schmid factor in the slip model of nt-Cu.

## 1. Introduction

Copper has been the most important conductor in microelectronic devices owing to its high ductility and conductivity. In recent years, it is discovered that by introducing nano-scale twins into the microstructure of copper, the mechanical strength, ductility, and electromigration resistivity can be improved, with nearly identical conductivity as bulk copper [1,2,3].

Nanotwinned copper is generally achieved in two ways—sputtering and electroplating deposition [2,4,5,6,7,8,9]. The advantage of sputtering is the high purity in the copper film, with the ability to control the preferred orientation of grains. Sputtered <111>-oriented nanotwinned copper is shown to have high thermal stability and strength [2,10]. Direct current electroplating is highly compatible with industrial mass production. Electroplated nanotwinned copper is classified into two groups—equiaxial grain nanotwinned copper and <111>-oriented nanotwinned copper. K. Lu et al. [1,8] have made many breakthroughs of the equiaxial grain nanotwinned copper, and has systematically conducted research on the effect of grain boundary and twin spacing on tensile strength. The equiaxial grain nanotwinned copper is different from the highly <111>-oriented nanotwinned copper. The latter showed strong anisotropic behavior in many properties owing to the highly <111> surface orientation.

Copper is also widely used as redistribution layers in the advanced packaging industry, such as integrated fan-out wafer level packages (InFO) [11]. In InFO structures, owing the presence of epoxy molding compound, interconnects can be redistributed on the outer regions of the chip to increase the bonding points. To connect these interconnects, copper wires must pass through the silicon and molding compound boundaries, causing a reliability issue. In 2017, researchers discovered that, when fan-out wafers underwent thermal cycling tests, thermal stress builds up owing to the difference of the material thermal coefficient, ultimately causing the copper wires to break [12]. Nanotwinned copper is shown to have ideal fatigue resistance. L. Lu et al. [13] have performed fatigue tests on nanotwinned copper, and discovered the interaction between high density twins and dislocations will not cause dislocation tanglement under repetitive stress. This is an example of the advantage of nanotwinned copper.

From the previous studies, we aim to apply nt-Cu in advanced packaging industries owing to its ideal mechanical properties. However, no suitable model has been proposed to explain the mechanical properties of highly <111>-oriented columnar nt-Cu. Therefore, we hope to come up with a model to clarify the relation between microstructure and its mechanical properties. With a newly proposed model, we can accurately modify the microstructure for the specific mechanical properties required.

## 2. Experimental

To conduct tensile tests, columnar nanotwinned copper with high elongation nearly all have had a film thickness of 100 μm [7,14,15]. In this study, we fabricate 20 μm thick foils, which is the thickness needed for redistribution layer (RDL), via direct current electroplating for mechanical tests. We altered the nanotwins’ microstructure by tuning the electroplating parameters and annealing temperature.

The plating substrates used in the experiment were Si wafers coated subsequently with a 5 μm thick polyimide layer, 100 nm thick Ti barrier layer, and 200 nm Cu seed layer. After sputtering the seed layer, the Si wafer was diced into 11 × 4 cm^2^ pieces for electroplating. The electroplating bath is made of highly purified CuSO_4_, with 50 g/L Cu ion; 40 ppm hydrochloric acid; 50 g/L sulfuric acid; and 0.4 ml/L commercial nt-Cu additives ECD-107A, ECD-107B, and ECD-108C provided by Chemleader corporation, Taiwan, China. Samples mentioned in this article electroplated with these different additives will henceforth be referred as samples type-A, type-B, and type-C, respectively. In this work, we modified the diameter and the twin spacing of the columnar nt-Cu grains with specific additives to understand the relationship between the microstructure and mechanical properties. The nt-Cu foils results are referred to later according to the additive used.

**Figure 1:**
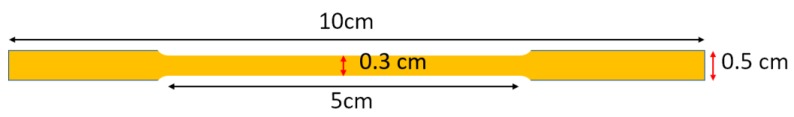
The schematic diagram of the nt-Cu specimen for tensile tests.

For electroplating, a high-purity soluble Cu target was placed on the anode side and the Si substrate was placed at the cathode side. Direct current electroplating was performed at room temperature, with a current density of 40 mA/cm^2^ (4ASD) and a stirring rate of 1200 rpm. After electroplating, electropolishing was applied to reduce the surface roughness of the nt-Cu foils. The electrolyte for electropolishing consisted of phosphoric acid, acetic acid, and glycerin. The electropolishing was performed at room temperature with a constant voltage of 1.75 V and operating time of 600 s. A platinum target was placed on the cathode side and the Cu foil was placed on the anode side during electropolishing. After electropolishing, the nt-Cu foils were peeled off from the Si substrate to be free-standing Cu foils. In addition, the type-B nt-Cu foils were annealed in N_2_ ambient at 200 °C, 300 °C, and 400 °C for 1 h to alter the average grain size and twin spacing. Finally, the nt-Cu foils were punched into a dog-bone shape, as depicted in Figure 1 for tensile tests, which were performed at room temperature with a strain rate of 4.17 × 10^−3^ l/s.

The grain orientation was observed by electron backscattered diffraction (EBSD) equipped in a JEOL JSM-7800F Prime Scanning Electronic Microscopy (SEM, JEOL JSM-7800F, Akishima, Tokyo, JAPAN), and the crystallographic texture and statistical orientation image maps were analyzed by Aztec EBSD post-processing software (Ver. 3.3, Oxford instruments, Abingdon, United Kingdom). The step size of EBSD ranged from 20 nm to 0.5 μm for different analysis areas. A minimum grain size of 50 nm and tolerance angle of 5° were set for grain size analysis. The low angle grain boundary was defined with a maximum misorientation angle of 15°. The misorientation angle of high angle grain boundary is larger than 15°. The tolerance angle of Σ3 twin boundary was set to be 1°.

A dual-beam focused ion beam system (TESCAN GAIA3) was employed to observe the cross-sectional microstructure.

## 3. Results and Discussions

### 3.1. Tuning of Microstructure With Different Additives During Electroplating

**Figure 2:**
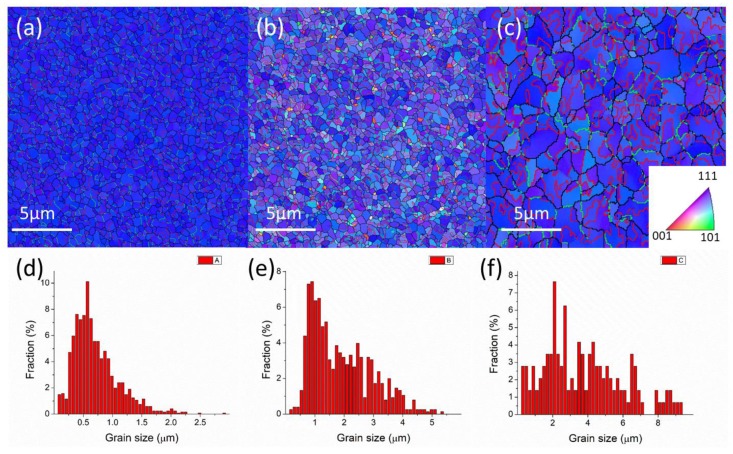
Plan-view grain orientation maps (OIMs) of highly <111>-oriented nt-Cu foils, which were deposited with additives (**a**) A, (**b**) B, and (**c**) C. The inverse pole figure in (**c**) indicated that the blue regions in the OIMs were <111>-oriented grains. The distribution of the columnar grain size of (**d**) type-A nt-Cu, (**e**) type-B nt-Cu, and (**f**) type-C nt-Cu.

**Figure 3:**
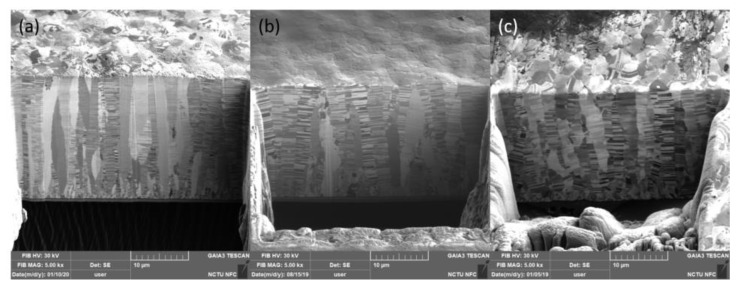
Cross-sectional ion images of (**a**) type-A, (**b**) type-B, and (**c**) type-C nt-Cu foils.

**Figure 4:**
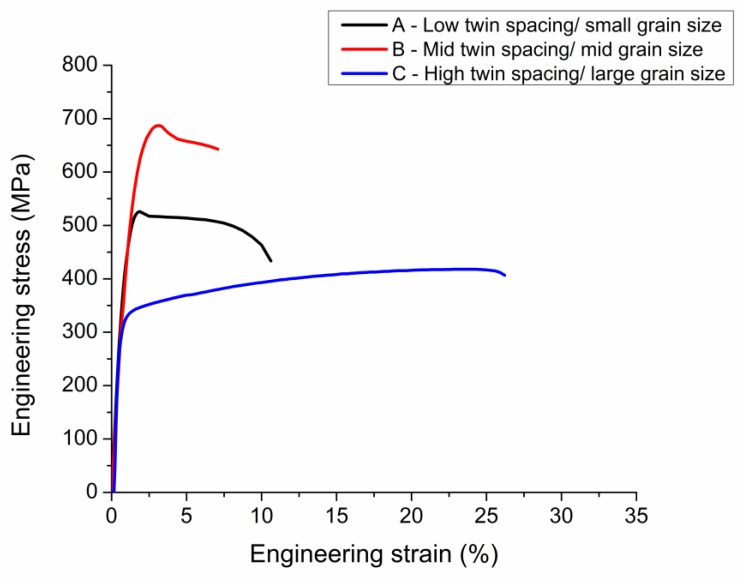
Stress–strain curves of the three types of <111>-oriented nano-twinned Cu foils. The yielding strength ranges from 297 to 698 MPa, and the elongation ranges from 6% to 26%.

The microstructure of the three types of nt-Cu foils before the tensile test is shown in Figure 2 and Figure 3. From the top-view grain orientation image maps, the Cu foils are all shown to be highly <111>-oriented. The areal fraction of grains with {111} surface is higher than 95%. In Figure 2a–c, the low angle grain boundary, high angle grain boundary, and twin boundary were marked with green lines, black lines, and red lines, respectively. In addition, most of the grain boundaries of the three types of nt-Cu are high angle grain boundaries. The grain size distributions of the three types of nt-Cu foils are shown in Figure 2d–f. In this study, the average grain size and twin spacing were modified in two different ways. First, by adjusting the components of the electroplating bath, the average grain size of nt-Cu foils type-A, type-B, and type-C was 1.1, 2.9, and 6 μm, respectively. The copper foils showed similar columnar grains with high density twinning structure in the cross-sectional ion images; see Figure 3. The average twin spacing of these nt-Cu foils was 31, 42, and 73 nm, respectively. The stress–strain curves of the nt-Cu foils are shown in Figure 4. The yielding strength has a large range, which could be attributed to the as-deposited microstructure of <111>-oriented nt-Cu. Type-A nt-Cu has the lowest twin spacing and the smallest grain size; type-B nt-Cu has mid twin spacing and mid grain size; and type-C nt-Cu has the largest twin spacing and grain size among the three types of nt-Cu. The stress-drop at the fracturing point was caused by serious necking. For type-A and type-B, an obvious necking point formed after the yielding point, and the elongation was dominated by the necking point. In contrast, the necking behavior is relatively uniform in the type-C Cu foil, the shrinkage in the width direction was observed through the gauge length. By comparing the grain size and twin spacing, we tuned the microstructure to obtain the desired mechanical properties.

Grain boundary strengthening is a well-known strengthening mechanism in materials. However, the high density twinned structure would limit the density and mobility of dislocation. The Hall–Petch equation explains the effect of grain size on yield strength; see Equation (1). Normally, for copper, the grain size needs to reach nanoscale to show an obvious increase in yield strength. However, in our experiment, we observed a yield strength of 698 MPa with grains of micron size. Therefore, we assume the traditional explanation of grain boundary strengthening is inadequate to cover nanotwinned copper owing to the presence of nanotwins.

(1) $$\sigma_{y}=\sigma_{0}+\frac{k_{y}}{\sqrt{d}}$$

$\sigma_{0}$ is the friction stress; $k_{y}$ is strengthening coefficient, and d is average grain diameter. The limiting mechanism of the interlayer thickness of multi-layer materials was derived in the confined layer slip (CLS) model [16]. Substituting the limiting thickness with twin spacing in this model can result in Equation (2).

(2) $$\sigma_{cls}=M\beta \frac{\mu b}{\lambda }\ln\frac{\alpha \lambda }{b}$$

where M is the Taylor factor, *μ* is the shear modulus, b is the Burger’s vector component, *λ* is the twin spacing, *α* is the material constant related to the dislocation core, and *β* is Poisson’s ratio.

**Figure 5:**
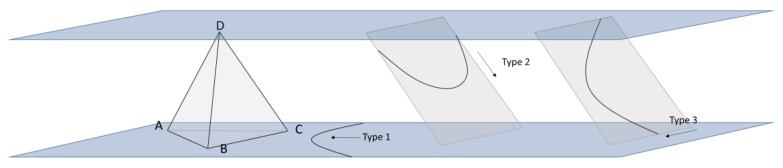
The schematic diagram of dislocation slip systems in nano-twinned Cu.

Taylor’s law could explain deformation in multi-slipping systems, yet grains in columnar <111>-oriented copper nanotwins have identical slip plane systems and similar slip behavior, along with the highly z-directional preferred orientation. In a highly <111>-oriented nanotwinned copper, if the {111} twin plane is defined as a normal (111) plane, it becomes harder to slip owing to Schmid’s factor. When the tensile axis is parallel to the coherent twin plane, the shear stress on the surface is 0 (the slip systems of dislocations between two coherent twin boundaries are shown in Figure 5). In face-centered-cubic (FCC), there are 12 individual slip systems that were components in 4 slip planes and with 3 slip directions for each slip plane. The slip systems between two twin boundaries could be divided into three types. In type 1, the slip plane and slip direction of the dislocation are parallel to the twin boundaries; in type 2, the slip plane and slip direction of the dislocation are both non-parallel to the twin boundaries; and in type 3, the slip plane is non-parallel to the twin boundaries, but the slip direction is parallel to the twin boundary.

**Figure 6:**
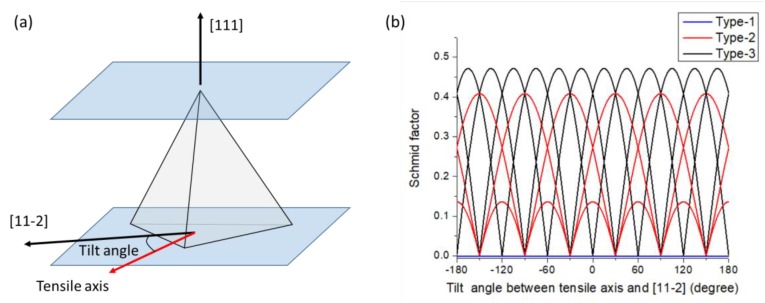
(**a**) The schematic diagram of the tensile axis of a <111>-oriented columnar nt-Cu grain during tensile test. (**b**) Schmid factor of the three types of slip systems in <111>-oriented columnar nt-Cu grains with different tilt angles between [$11\bar{2}$] and the tensile axis.

To clarify the main differences among the three types of slip systems, we defined the twin plane as the (111) plane in a columnar grain. In this study, the tensile axis is parallel to the twin plane, which means the tensile axis must be vertical to the normal twin plane (111). In this situation, we defined [11$\bar{2}$] vector as a reference direction of tensile axis and the rotation axis is defined as [111] (Figure 6a). We calculate the Shmid factor of the three types of slip systems. Figure 6b indicated that the Schmid factor of type 1 slip systems is 0, and the value of type 3 will always be higher than the value of type 2, except for some specific points with a tilt angle of 30° ± n × 60°. The directions with a tilt angle of 30° ± n × 60° were identified to be ±[$1\bar{1}0$], ±[$10\bar{1}$], and ±[$01\bar{1}$]. In other words, when the tensile axis is parallel to ±[$1\bar{1}0$], ±[$10\bar{1}$], and ±[$01\bar{1}$], Schmid factor of type 2 slip systems will be equal to the value of type 3. However, dislocations of the type 2 slip system need additional stress (0.8 GPa) to pass through the twin boundary [17]; that is, even if the Schmid factors are equal, dislocations still require more stress in the type 2 slip system than in the type 3 slip system to drive macroscopic deformation. The dislocation density results reported in previous studies support this theory [13,14,18].

Therefore, we can assume that there is only the type 3 slip system that has three individual slip systems in each highly <111>-oriented columnar nt-Cu grain. However, the assumption for Taylor’s theory required five independent slip systems in each grain, which means Taylor’s theory is unsuitable for the plastic deformation of columnar nt-Cu conducted in this study.

The main feature from Schmid law is the higher priority in deformation of harder grains over softer grains under tensile stress, even if the critical torque has been reached at lower stresses. There are only three viable slip planes in the highly-oriented nanotwinned copper. Under the circumstance of limited slip systems, while having the same (111) plane, the effect of the Schmid factor is not obvious. It is well known that when FCC structure slip systems are under tensile forces perpendicular to the <111> direction, the Schmid factor has a minimum value of $\frac{1}{\sqrt{6}}$ along the <$\bar{2}11$> and <$0\bar{1}1$> direction. This is nearly identical to the average Schmid factor value on the tensile direction that we have obtained in our EBSD analysis, that is, 0.45 ± 0.03. Therefore, in <111>-oriented nt-Cu, the slip system in every columnar grain is very similar, and under plastic deformation, the overall yield strength will be very similar owing to similar Schmid factors, which would reduce work hardening formed by dislocation entanglement, as shown in Figure 4. However, in order to observe this phenomenon clearly, a very small twin spacing is required.

Thus, we confirm that, on a highly <111>-oriented columnar nanotwinned copper, the columnar grain strength perpendicular to the tensile strength is identical in every grain. Therefore, the difference of critical torque between different grains needed to reach activation in plane slip becomes similar to single crystals. The Schmid factor for single crystals is much more suitable than the Taylor factor to explain the deformation in nanotwinned copper, despite that it is a polycrystalline system. Equation (3) is an alternation of the Schmid factor. The stress value represents different strengthening values under different twin densities.

In this experiment, the twin spacing is a variable. If we consider grain size along with twin spacing, we end up with Equation (4). The difference from previous literature is that all interfaces between different grain layers are fully connected, but we speculate from the microstructure observed by ion beam images that not all neighboring twin grains are fully aligned, despite them having a highly preferred orientation. The diameter of the columnar grains will affect the density of dislocation within the grains [19], and thus the effect of grain size on decreasing yield strength must be considered.

(3) $$\sigma_{cls}=\frac{\beta }{m}\frac{\mu b}{\lambda }\ln\frac{\alpha \lambda }{b}$$

(4) $$\sigma =\sigma_{0}+\frac{k_{y}}{\sqrt{d}}+\frac{\beta }{m}\frac{\mu b}{\lambda }\ln\frac{\alpha \lambda }{b}$$

The parameters are substituted into Equation (4) [7,16,19], where $\sigma_{0}$ is the friction stress = 25 MPa and K_y_ is the grain boundary strengthening coefficient. We implement the average values obtained from the EBSD analysis before the tensile tests, Schmid factor = 0.45, β = 0.218, and α = 0.16, and substitute the nanotwins’ twin spacing and grain diameter of all tested samples into Equation (4) to obtain the yield strength of nanotwinned copper A, B, and C to be 715 MPa, 548 MPa, and 374 MPa, respectively, which are very close to our experimental results.

### 3.2. Altering the Mechanical Properties of Nanotwinned Copper by Annealing 

**Figure 7:**
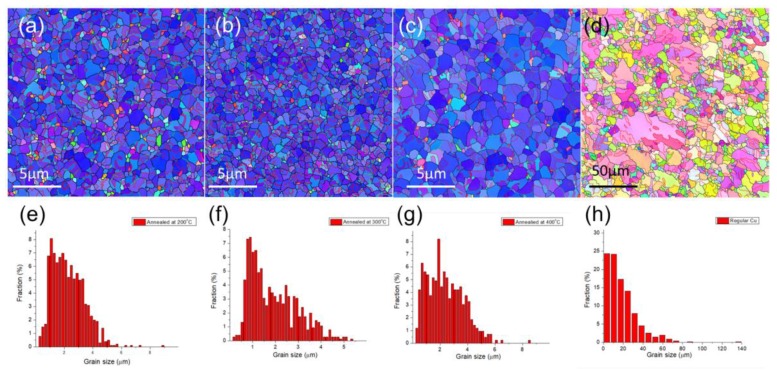
Plan-view grain orientation maps of B-type nt-Cu annealed at (**a**) 200 °C, (**b**) 300 °C, and (**c**) 400 °C for 1 h, and (**d**) 400 °C for 3 h. The low angle grain boundaries, high angle grain boundaries, and twin boundaries are marked in green lines, black lines, and red lines, respectively. (**e**) to (**h**) represent the grain size distribution of the specimen in (**a**) to (**d**). The surface preferred orientation transformed from <111> to random orientation after annealing at 400 °C for 3 h.

We studied the effect of various annealing temperatures on the B-type nanotwinned copper. Grain size was obtained from the EBSD results and twin spacing was calculated via cross-section ion beam image. As seen in Figure 7a–c, nanotwinned copper annealed at 200–400 °C for an hour does not show obvious grain growth, nor a change of its preferred orientation. As the annealing temperature increases, we can see from the EBSD images that the grain size also increases. The average grain size of samples annealed at 200 °C, 300 °C, and 400 °C for 1 h is 2.95, 3.3, and 3.7 μm, respectively. The average grain size of samples annealed at 400 °C for 3 h has a diameter of 18.4 μm.

**Figure 8:**
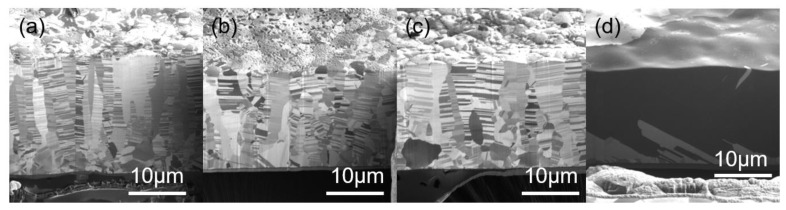
Cross-sectional ion images of the type-B nt-Cu after annealing at (**a**) 200 °C, (**b**) 300 °C, and (**c**) 400 °C for 1 h, and (**d**) 400 °C for 3 h.

**Figure 9:**
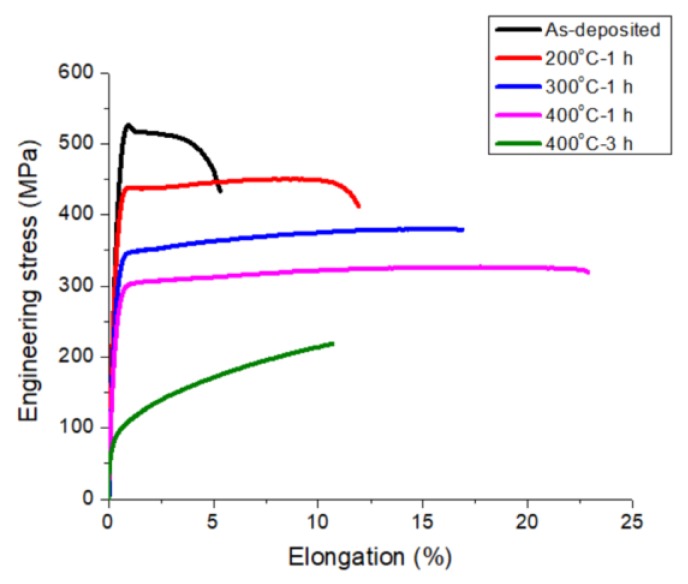
Stress–strain curves of the type-B nt-Cu foils before and after annealing at 200 °C, 300 °C, and 400 °C for 1 h, and 400 °C for 3 h.

The ion beam images of annealed samples at various temperatures are shown in Figure 8. From Figure 7 and Figure 8, we can observe the trend of increasing grain diameter and twin spacing after annealing. Columnar <111>-oriented nanotwinned copper was proven to have great thermal stability. In this study, it is able to withstand annealing at 400 °C for 1 h and maintain its preferred orientation. On the other hand, after annealing at 400 °C for 3 h, the surface of the nanotwinned structure will lose its <111> orientation and transform into randomly oriented large grains. From the cross-section images, we can no longer observe twinned grains parallel to the surface after annealing, instead a few micron-scaled annealed grains that can be viewed as large copper grains. From the cross-section ion beam image, we are able to calculate the twin spacing, which is 55, 82, and 98 nm for samples annealed at 200 °C, 300 °C, and 400 °C, respectively. The strain–stress curve from the tensile tests is shown in Figure 9, where we observe that, with the increasing annealing temperature, there will be an increase in ductility, but with a decrease in yield strength. The increase in ductility continues until the microstructure undergoes a massive change, when the high twin density disappears or when there is no longer a preferred orientation. The copper film will then show a decrease in ductility, and we can observe a decrease in yield strength and obvious work hardening.

From the conclusion in the last section, we substitute the microstructure parameters from the post-annealing results into Equation (4) to obtain the theoretical yield strength. The value is 462, 356, and 317 MPa for samples annealed at 200 °C, 300 °C, and 400 °C for 1 h, respectively, which is very close to the results obtained in the tensile tests. Concerning the large copper grains, as there are no nano-scale twins or layer-like structure to hinder its dislocation slip, the CLS model is not suitable. However, we can assume from the tensile curves in Figure 9 that, without the presence and the limitations of dislocation slip interfaces, the dislocations can entangle during slip, causing a notable work hardening. Besides, as the entanglement of dislocation causes discontinuity of dislocation slip, we observed, in the 400 °C for 3 h sample, an elongation decrease of 10%, roughly half of the 400 °C for 1 h sample or the type-C sample from Figure 4. The parameters of the annealed nt-Cu foils are substituted into Equation (4), and the predicted yield strengths for as-deposited, 200 °C, 300 °C, and 400 °C annealing for 1 h are 548 MPa, 464 MPa, 364 MPa, and 326 MPa, respectively. The experimental results are very close to the theoretical values.

**Figure 10:**
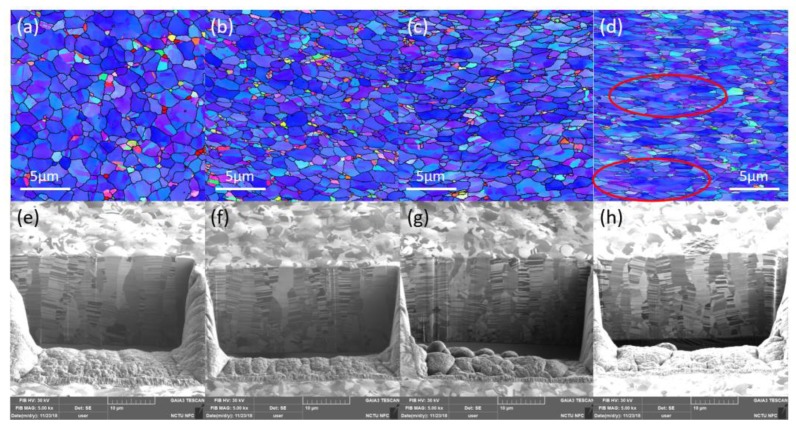
The top-view OIMs of the type-B nt-Cu at (**a**) ε_x_ = 4%, (**b**) ε_x_ = 11%, (**c**) ε_x_ = 23%, and (**d**) ε_x_ = 32%, where the ε_x_ is defined as the strain along tensile direction. (**e**) to (**f**) are the correspond to cross-sectional ion images of the specimen in (**a**) to (**d**).

We observed the microstructures of type-B copper foils annealed at 400 °C with different deformation scenarios, as shown in Figure 10. From the EBSD results, we observed an obvious elongation of the <111> columnar grains, and the unchanged structure of non-<111> grains from Figure 10a–d. This implies that, during the deformation process, the strain rates between different grains are uneven. Elongation is mainly dependent on the strain rate of the columnar grain under tensile stress.

Besides the above discussed strengthening model, we also observed an increase in elongation with the decrease in sample strength, which corresponds well to previous studies [7]. However, in previous studies, as the deformation increases, many sub-grains or dislocation cells appear on the boundaries of the columnar nanotwinned copper grains. It is not observed in this study, from ion beam images of the cross-section microstructure (Figure 10a–h), we can see that no sub-grains were present. Instead, we can observe, from the surface EBSD analysis, an increase in average grain size, where grains are able to reach up to 15 μm in diameter. This suggests that nanotwinned copper undergoes grain growth under tensile strength, which has not been reported in previous studies. In addition, in other columnar twin copper foil studies [2,9,20], elongation rates are commonly not ideal, plus the fact that thickness will greatly affect the elongation rates. It is rare to observe an elongation rate of over 25% with a thickness thinner than 20 μm.

## 4. Conclusions

In this study, we fabricated highly <111>-oriented nanotwinned copper foils via direct current electroplating. We altered the microstructure of the films by tuning the electroplating solution and annealing temperature under nitrogen atmosphere. After varying twin spacing and average grain size, and combining the confined layer slip with the Hall–Petch equation, we calculate the theoretical yield strength, which matches well with the experiment results. Owing to the unique crystal orientation properties of <111>-oriented columnar grains, dislocations entanglement caused by slip is very limited. The Schmid factor of grains along the tensile axis direction is highly identical, and thus the explanation of the plastic deformation is more suitable using the Schmid factor model. Thus, we replace the Taylor factor with the Schmid factor in the confined layer slip model. We also observed unprecedented microstructure evolution without sub-grains formation, but rather a grain growth phenomenon. We observed a yield strength range of 300–700 MPa and elongation rate range of 5%–25% for <111>-oriented nanotwinned copper, which shows the potential of applications in RDL in electronic packaging technology.

## References

[1] Lu L., Shen Y., Chen X., Qian L., Lu K. (2004). Ultrahigh Strength and High Electrical Conductivity in Copper. Science.

[2] Zhang X., Wang H., Chen X.H., Lu L., Lu K., Hoagland R.G., Misra A. (2006). High-Strength sputter-Deposited Cu foils with preferred orientation of nanoscale growth twins. Appl. Phys. Lett..

[3] Chen H.-P., Huang C.-W., Wang C.-W., Wu W.-W., Liao C.-N., Chen L.-J., Tu K.-N. (2016). Optimization of the nanotwin-Induced zigzag surface of copper by electromigration. Nanoscale.

[4] Huang Y.S., Liu C.M., Chiu W.L., Chen C. (2014). Grain growth in electroplated (1 1 1)-Oriented nanotwinned Cu. Scr. Mater..

[5] Liu T.-C., Liu C.-M., Hsiao H.-Y., Lu J.-L., Huang Y.-S., Chen C. (2012). Fabrication and Characterization of (111)-Oriented and Nanotwinned Cu by Dc Electrodeposition. Cryst. Growth Des..

[6] Hsiao H.Y., Liu C.M., Lin H.W., Liu T.C., Lu C.L., Huang Y.S., Chen C., Tu K.N. (2012). Unidirectional growth of microbumps on (111)-Oriented and nanotwinned copper. Science.

[7] You Z.S., Lu L., Lu K. (2011). Tensile behavior of columnar grained Cu with preferentially oriented nanoscale twins. Acta Mater..

[8] Lu L., Chen X., Huang X., Lu K. (2009). Revealing the Maximum Strength in Nanotwinned Copper. Science.

[9] Hodge A.M., Wang Y.M., Barbee T.W. (2008). Mechanical deformation of high-Purity sputter-Deposited nano-Twinned copper. Scr. Mater..

[10] Anderoglu O., Misra A., Wang H., Zhang X. (2008). Thermal stability of sputtered Cu films with nanoscale growth twins. J. Appl. Phys..

[11] Tseng C., Liu C., Wu C., Yu D. InFO (Wafer Level Integrated Fan-Out) Technology. Proceedings of the 2016 IEEE 66th Electronic Components and Technology Conference (ECTC).

[12] Yu C.K., Chiang W.S., Liu N.W., Lin M.Z., Fang Y.H., Lin M.J., Lin B., Huang M. A Unique Failure Mechanism Induced by Chip to Board Interaction on Fan-Out Wafer Level Package. Proceedings of the 2017 IEEE International Reliability Physics Symposium (IRPS).

[13] Pan Q., Zhou H., Lu Q., Gao H., Lu L. (2017). History-Independent cyclic response of nanotwinned metals. Nature.

[14] Cheng Z., Zhou H., Lu Q., Gao H., Lu L. (2018). Extra strengthening and work hardening in gradient nanotwinned metals. Science.

[15] Cheng Z., Lu L. (2019). The effect of gradient order on mechanical behaviors of gradient nanotwinned Cu. Scr. Mater..

[16] Misra A., Hirth J.P., Hoagland R.G. (2005). Length-Scale-Dependent deformation mechanisms in incoherent metallic multilayered composites. Acta Mater..

[17] Liu Y., Jian J., Chen Y., Wang H., Zhang X. (2014). Plasticity and ultra-Low stress induced twin boundary migration in nanotwinned Cu by in situ nanoindentation studies. Appl. Phys. Lett..

[18] Pan Q.S., Lu Q.H., Lu L. (2013). Fatigue behavior of columnar-Grained Cu with preferentially oriented nanoscale twins. Acta Mater..

[19] Ye J.C., Wang Y.M., Barbee T.W., Hamza A.V. (2012). Orientation-Dependent hardness and strain rate sensitivity in nanotwin copper. Appl. Phys. Lett..

[20] Hodge A.M., Furnish T.A., Navid A.A., Barbee T.W. (2011). Shear band formation and ductility in nanotwinned Cu. Scr. Mater..

